# Colorectal cancer survival rates in Ghana: A retrospective hospital-based study

**DOI:** 10.1371/journal.pone.0209307

**Published:** 2018-12-19

**Authors:** Francis Agyemang-Yeboah, Joseph Yorke, Christian Obirikorang, Emmanuella Nsenbah Batu, Emmanuel Acheampong, Emmanuel Amankwaa Frimpong, Enoch Odame Anto, Bright Amankwaa

**Affiliations:** 1 Department of Molecular Medicine, School of Medical Science, Kwame Nkrumah University of Science and Technology (KNUST), Kumasi, Ghana; 2 Department of Surgery, Komfo Anokye Teaching Hospital, Kumasi, Ghana; 3 School of Medical and Health Sciences, Edith Cowan University, Joondalup, Western Australia, Australia; 4 Oncology Unit, Komfo Anokye Teaching Hospital, Kumasi, Ghana; University of Kwazulu-Natal, SOUTH AFRICA

## Abstract

**Background:**

Colorectal cancer (CRC) is one of the commonest cancers associated with diverse prognosis times in different parts of the world. Despite medical interventions, the overall clinical outcomes and survival remains very poor for most patients in developing countries. This study therefore investigated the survival rate of colorectal cancer and its prognostic factors among patients at Komfo Anokye Teaching Hospital, Ghana.

**Methodology:**

In this retrospective cohort study, a total of 221 patients diagnosed with CRC from 2009 to 2015 at the Surgical and Oncological units of Komfo Anokye Teaching Hospital (KATH), Kumasi, Ghana were employed. The survival graphs were obtained using the Kaplan–Meier method and compared by the Log-rank test. Cox regression analysis was used to assess prognostic factors. All analyses were performed by SPSS version 22.

**Results:**

The median survival time was 15 months 95% CI (11.79–18.21). The overall survival rate for CRC over the 5 years period was 16.0%. The survival rates at the 1^st^, 2^nd^, 3^rd^, 4^th^ and 5^th^ years were 64% 95% CI (56.2–71.1), 40% 95% CI (32.2–50.1), 21% 95% CI (11.4–30.6) 16% 95% CI (8.9–26.9) and 16% 95% CI (7.3–24.9). There was a significant difference in the survival rate of colorectal cancer according to the different stages (p = 0.0001). Family history [HR = (3.44), p = 0.029)], Chemotherapy [HR = (0.23), p = <0.0001)], BMI [HR = (1.78), p = 0.017)] and both chemo/radiotherapy (HR = (3.63), p = 0.042)] were the significant social and clinical factors influencing the overall survival. Pathological factors such as TNM tumour stage (p = 0.012), depth of tumour invasion (p = 0.036), lymph node metastasis (p = 0.0001), and distance metastasis (p = 0.001) were significantly associated with overall survival.

**Conclusion:**

The study has clearly demonstrated that survival rate for CRC patients at KATH, Ghana is very low in a 5 years period. This is influenced by significant number of clinical and pathological prognostic factors. Identification of prognostic factors would be a primary basis for early prediction and treatment of patients with colorectal cancer.

## Introduction

Globally, colorectal cancer is one of the commonest cancers and in the western countries; it is the second leading cause of cancer mortality. [1]. The difference in survival rates observed in various clinical trials maybe due to the variations in patient’s characteristics and prognostic factors [2]. Survival of colorectal cancer has improved dramatically over the last decade as a result of the invention of new drugs and targeted therapies. [3]. However, enormous disparities in colorectal cancer survival exist within regions and across the global [4, 5]. These differences are not easily understood, although most of the disparities in CRC survival can be attributed to variation in the accessibility to treatment and diagnostics [5]. In addition, molecular analyses performed so far indicates that the pathogenesis of all CRCs varies at different stage tumours. Even for individual patients with same stage tumours, response to treatment and long term prognosis varies[6].

Over the past years, several research groups have suggested numerous factors associated with the survival of CRC patients.[7, 8]. However, the extent of tumour infiltration to the bowel wall, adjacent lymph node metastases and distant metastasis are the major contributing factors [9]. Although, various studies [10, 11] have reported a strong correlation between colorectal cancer stage and its prognosis, it has also been argued in other studies [10, 12] that the prognosis for a patient with colorectal cancer is much influenced by factors relating to patients characteristics and the tumour but not just the anatomical extension of the tumour.

Additionally, other studies have also showed that the initial treatment administered, body mass index (BMI), marital status, tumour grade, tumour size and pathologic stage of tumour are significantly associated with the survival of CRC patients [5, 13]. Recent studies have shown that the survival of CRC in sub-Saharan African is very low due to late presentations and lack of modern specialized systems for treatment [14]. In Ghana, the number of new cases of colorectal cancer has increased by 8- fold per year from an average of 4.1 new cases in 1960s to an average of 32.6 new cases currently [15, 16]. In 2010, Dakubo *et al* reported a crude incidence rate of 11.18 per 100,000 populations in both sexes. Moreover, Laryea et al., (2014) reported a crude incidence and age standardized incidence of 0.1 and 0.3 per 100,000 population [17]. Some studies have identified other factors such as helicobacter pylori infection, the dietary component of red meat, beef, lamb, pork and veal and its processed varieties as the predominant risk factors in Ghana [16, 18, 19]. There is paucity of data on the survival rate of CRC as well as its associated factors in Ghana. Knowledge of prognostic factors in our population will be the foundation for planning treatment and predicting the outcome of patients with colorectal cancer. It is thus, against this background that this study investigated the survival rate of colorectal cancer and its prognostic factors among patients at Komfo Anokye Teaching Hospital, Ghana.

## Methodology

### Study design/setting

This was a retrospective cohort study, conducted among CRC patients at the Surgical and Oncological (S&O) Department of the Komfo Anokye Teaching Hospital. Komfo Anokye Teaching Hospital (KATH) is the second largest and a referral teaching hospital located in Kumasi, the regional capital of the Ashanti region in Ghana. The region has an average total population of 4,780,380 (Ghana Statistical Service, 2010).

### Study population and participants’ selection

A total of 221 cases of CRC, recorded from 2009 to 2015 were retrospectively retrieved from the medical records of the S & O Department database with a 100% rate of accuracy. Information on socio-demographics characteristics, clinical and pathological variables including histological type, grade of tumour and TNM staging were recorded. Data on type of treatments was also reviewed. Moreover, BMI based on the patient’s current recorded weight and height since been diagnosed was also calculated. Smoking history comprised of patients who indicated that, they had ever smoked or was currently smoking, and alcohol intake also refers to patients who were currently alcoholics and those who used to be alcoholic.

### Inclusion criteria

Records showing complete clinical examination, indicating the presence of malignant tumour in the large bowel were included.

### Exclusion criteria

Patients with other large bowel conditions and histopathological confirmed non-malignant tumours were excluded.

### Follow-up

Patients were contacted during their follow-up visits to the hospital and those who could not report for review in the hospital were contacted via telephone. Deaths of subjects were confirmed via contact with their families and relatives. Survival periods were calculated from the date of diagnosis to the date of either last follow-up or death. Patients alive at the end of the follow-up and those lost to follow-up were censored either at the last contact or at death.

**Fig 1:**
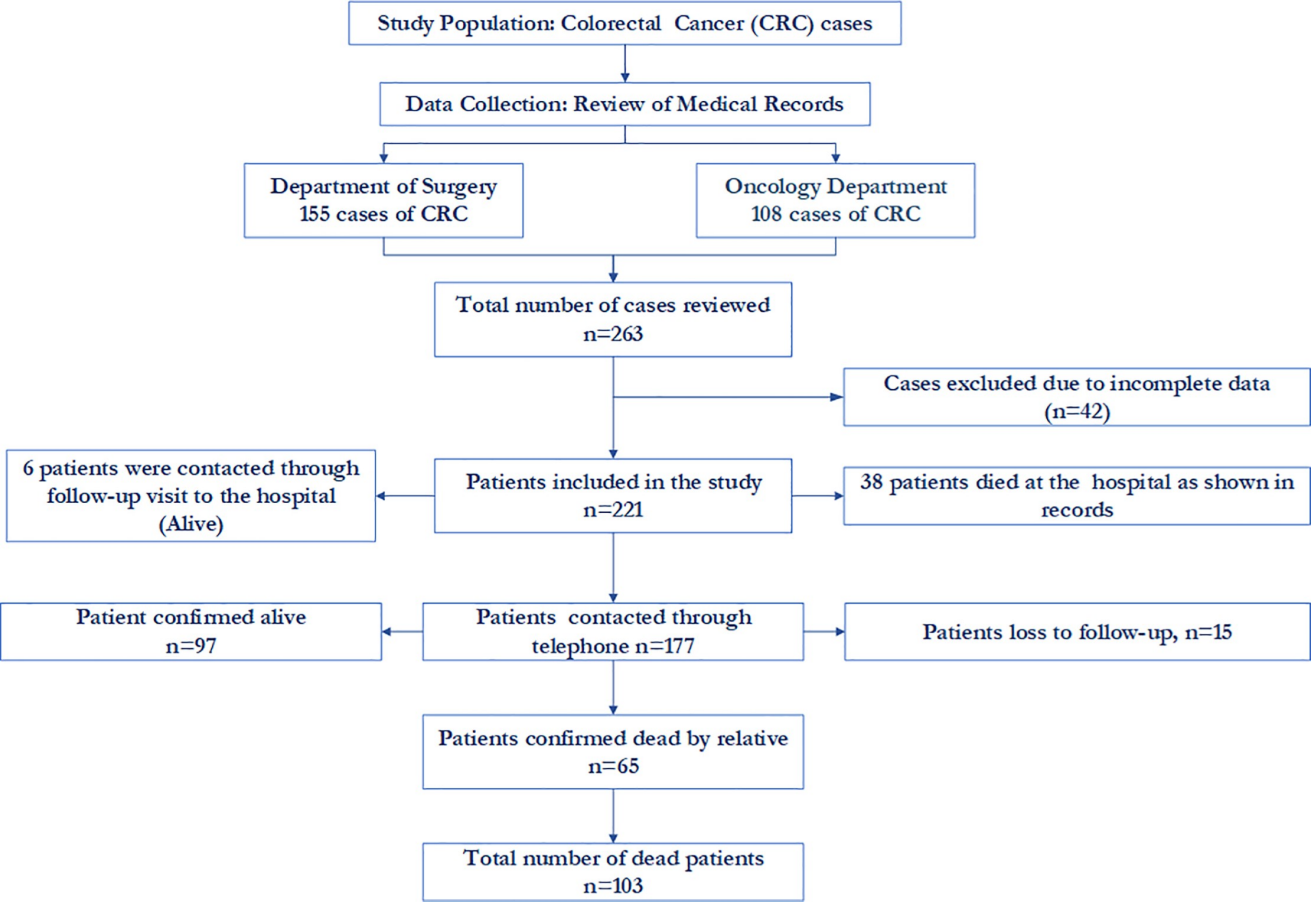
Procedure for the selection of cases for the study.

Fig 1 shows the procedure for the selection of cases for the study.

### Statistical analysis

Data entry and analysis were performed using SPSS version 20. Survival analysis was done using Kaplan–Meier method and the differences in patient survival periods were determined by employing the log-rank test in relation to socio-demographic and lifestyle characteristics, and clinical and pathological parameters [**Tables 1 and 2**]. To determine the prognostic factors for survival, all variables were tested for their relationship in the Cox-regression model. Proportional hazard (PH) assumptions were initially tested for each model [**Tables 3–5**] based on the scaled Schoenfeld residuals. The PH test was not significant for each of the covariates in each model and the global Schoenfeld test (GST) was not statistically significant for each model [Table 3; GST: p = 0.481, Table 4; GST: p = 0.186, Table 5; GST: p = 0.216], we therefore assumed the proportional hazards. Multicollinearity test was done for covariates in each model, and the variation inflation factor (VIF) value obtained for covariates in each model was within a range of 1–5 suggesting that there is no multicollinearity. Multivariable Cox regression analysis was carried using the force entry procedure. The Chi-square value obtained for the regression model for Table 4 (χ^2^ = 42.7, p<0.0001) and Table 5 (χ^2^ = 28.8, p = 0.017) were statistically significant, however Chi-square value for regression model for Table 3 did not show significance (χ^2^ = 15.8, p = 0.328). There were no statistically significant differences between patients who were follow-up and those who were not followed in relation to socio-demographic and lifestyle characteristics, clinical and pathological parameters [**S1 Table**]. A p < 0.05 was accepted as statistically significant.

**Table 1:** Association of socio-demographic and lifestyle characteristics with survival using log rank test.

Variable	Frequency (n, %)	Median OS (95% CI) (months)	P-value
**Age (years)**			0.241
< 40	40(22.6%)	28(3.5–52.5)	
40–49	31(14.0%)	17(5.0–29.0)	
50–59	58(26.2%)	14(6.6–21.4)	
60–69	43(19.5%)	14(11.5–16.5)	
≥70	39(17.6%)	20(9.3–30.7)	
**Gender**			0.996
Female	94(42.5%)	15(10.9–19.1)	
Male	127(57.5%)	14(8.6–18.2)	
**Marital Status**			0.464
Single	27(12.2%)	28(0.7–55.3)	
Married	140(63.3%)	14(11.0–16.9)	
Divorced	21(9.50%)	19(17.5–20.5)	
Widowed	33(14.9%)	15(1.8–28.2	
**Family History**			**0.036**
No	205(92.8%)	15(11.5–18.4)	
Yes	16(7.2%)	—	
**Presence of Comorbidities**			0.250
No	166(75.1%)	15(11.6–18.4)	
Yes	55(24.9%)	15(5.5–24.5)	
**Hypertension**			0.232
No	177(80.1%)	17(13.3–20.7)	
Yes	44(19.9%)	14(10.0–18.0)	
**Diabetes**			0.588
No	203(91.9%)	15(11.5–18.5)	
Yes	18(8.1%)	28(16.3–39.7)	
**Alcoholic intake**			0.717
No	200(90.5%)	15(11.6–18.4)	
Yes	21(9.5%)	21(5.8–36.2)	
**Smoking history**			
No	210(95.0%)	15(11.9–19.1)	0.696
Yes	11(5.0%)	13(10.6–15.4)	

OS = Overall Survival.

**Table 2:** Association of clinical and pathological parameters with survival using log rank test.

Variable	Frequency (n, %)	Median OS (95%CI) (Months)	P-value	Variables	Frequency (n, %)	Median OS (95%CI) (Months)	P-value
**Duration of Symptoms (months)**			0.567	**Tumour Location**			0.405
< 6	95(43.0%)	19(13.4–24.6)		Colon	75(33.9%)	14(7.3–20.7)	
6 to 12	85(38.5%)	14(11.6–16.4)		Rectum	108(48.9%)	17(11.2–22.8)	
> 12	41(18.6%)	14(10.2–17.7)		Anorectum	18(8.1%)	18(0.8–37.9)	
**Surgery**			0.640	Anal	13(5.9%)	12(10.6–13.4)	
No	76(34.4%)	15(9.9–20.1)		More than one site	7(3.2%)	19(6.6–31.4)	
Yes	145(65.6%)	14(8.8–19.2)		**Histological Grade**			0.332
**Nature of Operation**			0.741	Well differentiated	51(23.1%)	14((0.9–29.8)	
Emergency	49(33.8%)	14(8.8–19.2)		Moderately differentiated	104(47.1%)	15(11.0–18.9)	
Elective	96(66.2%)	19(12.6–25.4)		Poorly differentiated	25(11.3%)	30(0.4–73.9)	
**Chemotherapy**			**0.0001**	Undifferentiated	41(18.6%)	18(15.6–20.4)	
No	118(53.4%)	11(8.0–14.0)					
Yes	103(46.6%)	30(18.0–42.0)		TNM Tumour Stage			**0.0001**
**Radiotherapy**			0.402	Stage I	13(6.0%)	48(41–56.5)	
No	166(75.1%)	14(9.8–18.2)		Stage II	64(29.0%)	36 (15.2–56.7)	
Yes	55(24.9%)	17(13.1–20.8)		Stage III	89(40.3%)	14 (11.1–16.9)	
**Chemo-radiotherapy**			0.587	**Stage IV**	55(24.9%)	11(6.0–16.0)	
No	173(80.1%)	15(11.0–19.0)					
Yes	43(19.9%)	15(11.8–18.2)		Lymph Node Metastasis			**0.0001**
**BMI Categories**			**0.036**	N0	78(35.3%)	36(21.4–50.6)	
Underweight	77(34.8%)	11(5.3–16.7)		N1	88(39.9%)	12(9.5–14.5)	
Normal	90(40.7%)	18(9.7–26.3)		N2	55(24.9%)	15(10.1–19.9)	
Overweight	32(14.5%)	26(15.2–36.8)		Distant Metastasis			**0.0001**
Obese	22(10.0%)	23(11.3–34.7)		M0	68(21.7%)	19(12.0–25.9)	
Depth of Tumour Invasion				M1	173(78.3%)	11(6.0–15.9)	
T2	18(8.2%)	28(15.1–40.9)					
T3	84(38.0%)	14(5.2–22.8)					
T4	119(53.8%)	14(10.7–18.7)					

OS = Overall Survival, BMI = Body Mass Index, P<0.05 = statistically significant.

**Table 3:** Association of socio-demographics and lifestyle characteristics with survival using Cox regression analysis.

	Univariate Analysis			Multivariate Analysis		
Variables	HR	95% CI	P-value	HR	95% CI	P-value
**Age**						
< 40	1					
40–49	0.51	(0.20–1.30)	0.160	0.49	(0.18–1.34)	0.159
50–59	1.28	(0.71–2.33)	0.424	1.27	(0.63–2.54)	0.520
60–69	1.30	(0.71–2.37)	0.399	1.36	(0.63–2.92)	0.465
≥70	1.16	(0.61–2.21)	0.655	0.97	(0.42–2.20)	0.909
**Gender**						
Female	1					
Male	1.00	(0.67–1.49)	0.996	1.11	(0.71–1.74)	0.626
**Marital Status**						
Single	1					
Married	0.91	(0.46–1.84)	0.799	0.94	(0.40–2.21)	0.877
Divorced	0.75	(0.37–1.51)	0.423	0.90	(0.43–1.89)	0.781
Widowed	**1.37**	(0.80–2.33)	0.25	**1.27**	(0.68–2.37)	0.439
**Family History**						
No	1					
Yes	2.73	(1.00–7.44)	**0.049**	3.44	(0.09–0.88)	**0.029**
**Presence of Comorbidities**						
No	1					
Yes	1.27	(0.84–1.94)	0.262	1.29	(0.74–2.26)	0.734
**Hypertension**						
No	1					
Yes	1.31	(0.83–2.08)	0.244	1.11	(0.45–2.75)	0.734
**Diabetes**						
No	1					
Yes	1.17	(0.65–2.10)	0.597	0.74	(0.35–1.55)	0.512
**Alcoholic intake**						
No	1					
Yes	0.88	(0.42–1.81)	0.722	0.65	(0.25–1.69)	0.401
**Smoking history**						
No	1					
Yes	0.84	(0.34–2.06)	0.703	1.40	(0.42–4.69)	0.607

HR = Hazard Ratio, CI = Confidence Interval, P<0.05 = statistically significant.

**Table 4:** Association of clinical parameters with survival using cox regression analysis.

	Univariate Analysis			Multivariate Analysis		
Variables	HR	95% CI	P-value	HR	95% CI	P-value
**Duration of Symptoms**						
< 6	1					
6–12	1.18	(0.671–2.057)	0.573	1.23	(0.78–1.93)	0.380
> 12	1.34	(0.759–2.379)	0.311	1.23	(0.62–2.26)	0.491
**Surgery**						
No	1					
Yes	1.11	(0.72–1.69)	0.648	3.82	(0.16–91.51)	0.408
**Nature of Operation**						
Emergency	0.99	(0.63–1.58)	0.985	3.41	(0.14–82.49)	0.451
Elective	1					
**Chemotherapy**						
No	1					
Yes	0.38	(0.25–0.57)	**0.0001**	0.23	(0.13–0.41)	**<0.0001**
**Radiotherapy**						
No	1					
Yes	0.82	(0.51–1.32)	0.413	0.56	(0.20–1.60)	0.282
**Both Chemo and Radiotherapy**						
No	1					
Yes	0.87	(0.52–1.46)	0.596	3.63	(1.05–12.59)	**0.042**
**BMI Categories**						
Normal	1					
Underweight	1.74	(1.11–2.72)	**0.016**	1.78	(1.11–2.86)	**0.017**
Overweight	0.95	(0.51–1.75)	0.860	0.93	(0.48–1.78)	0.817
Obese	0.94	(0.46–1.89)	0.852	0.95	(0.46–1.98)	0.894

HR = Hazard ratio, CI = confidence interval, BMI = body mass Index, P<0.05 = statistically significant.

**Table 5:** Association of pathological parameters with survival using cox regression analysis.

	Univariate Analysis			Multivariate Analysis		
Variables	HR	95% CI	P-value	HR	95% CI	P-value
**Tumour Location**						
Colon	1					
Rectum	0.99	(0.64–1.53)	0.965	0.86	(0.50–1.48)	0.592
Anorectum	0.41	(0.14–1.14)	0.088	0.40	(0.12–1.37)	0.144
Anal	1.12	(0.49–2.5)	0.79	2.15	(0.69–6.62)	0.183
More than one site	1.28	(0.49–3.28)	0.612	0.76	(0.22–2.64)	0.660
**Histological Grade**						
Well differentiated	1					
Moderately differentiated	1.62	(0.94–2.80)	0.085	1.33	(0.70–2.52)	0.377
Poorly differentiated	1.58	(0.77–3.55)	0.229	1.01	(0.35–2.91)	0.986
Undifferentiated	1.66	(0.75–3.33)	0.193	1.56	(0.63–3.85)	0.338
**Tumour Stage**						
Stage 1	1					
Stage II	4.12	(0.55–30.84)	0.168	2.00	(0.16–25.79)	0.595
Stage III	9.41	(1.29–68.58)	**0.027**	4.97	(0.28–87.64)	0.274
Stage IV	12.89	(1.74–95.24)	**0.012**	13.34	(0.49–359.01)	0.123
**Depth of Tumour Invasion**						
T2	1					
T3	3.42	(1.05–11.11)	**0.041**	1.67	(0.37–7.61)	0.508
T4	3.51	(1.09–11.33)	**0.036**	1.93	(0.43–8.59)	0.389
**Lymph Node Metastasis**						
N0	1					
N1	2.65	(1.58–4.43)	**0.0001**	1.01	(0.25–4.08)	0.991
N2	2.42	(1.24–4.73)	**0.009**	0.71	(0.17–2.95)	0.641
**Distant Metastasis**						
M0	1					
M1	2.16	(1.37–3.40)	**0.001**	0.49	(0.11–2.18)	0.352

HR = Hazard Ratio, CI = confidence Interval, T = tumour depth, N = lymph node metastasis, M = distant metastasis, P<0.05 = statistically significant

### Ethical consideration

Ethical Approval for the study was obtained from the Committee on Human Research, Publication and Ethics (CHRPE/AP/286/15) of the School of Medical Sciences (SMS), Kwame Nkrumah University of Science and Technology (KNUST) as well as the Research and Development (R&D) Unit of the KATH.

## Results

**Fig 2:**
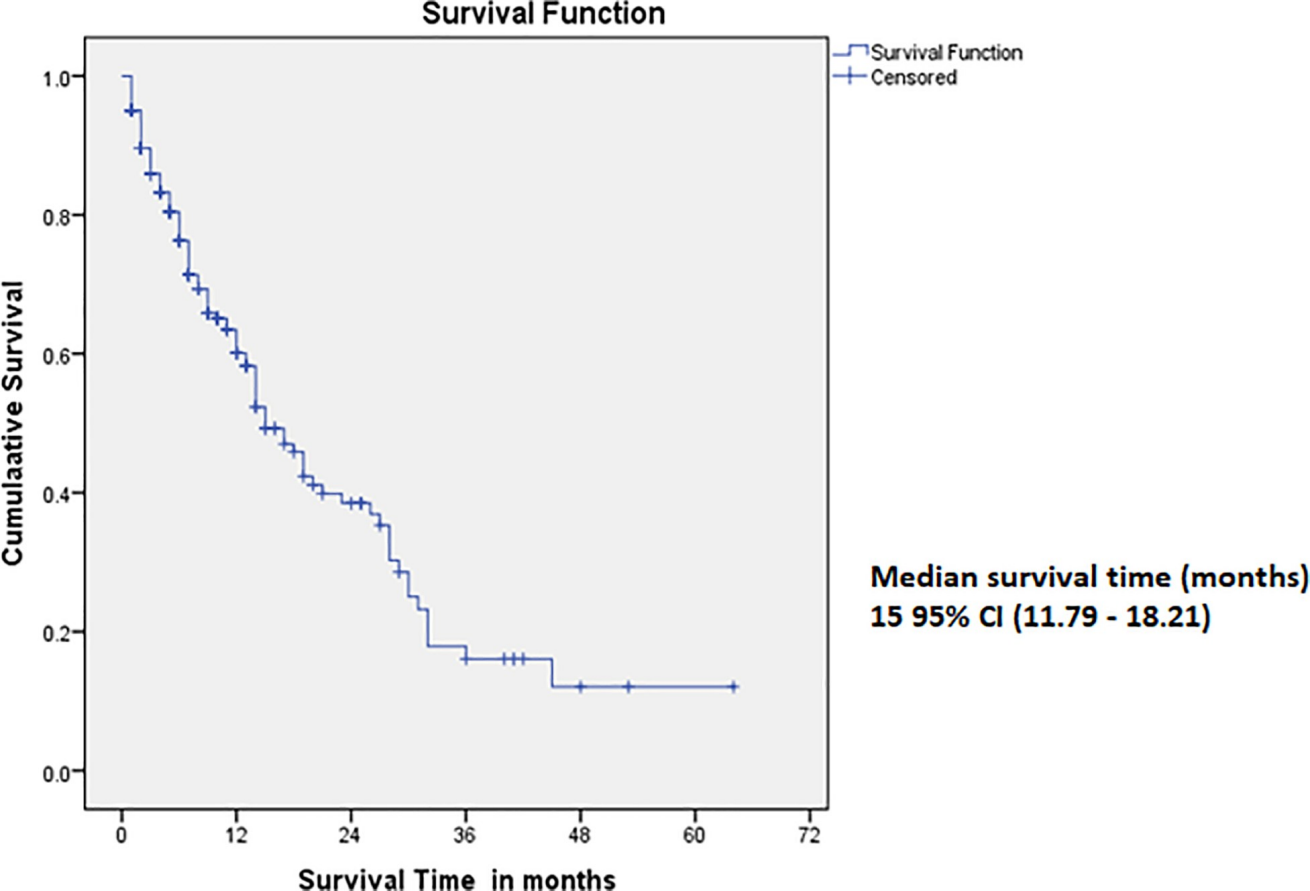
Overall 5 year’s survival function curve in colorectal cancer patients at KATH.

As shown in Fig 2, the median survival time was 15 months 95% CI (11.79–18.21). The survival rates at the 1^st^, 2^nd^, 3^rd^, 4^th^ and 5^th^ years were 64% 95% CI (56.2–71.1), 40% 95% CI (32.2–50.1), 21% 95% CI (11.4–30.6) 16% 95% CI (8.9–26.9) and 16% 95% CI (7.3–24.9).

**Fig 3:**
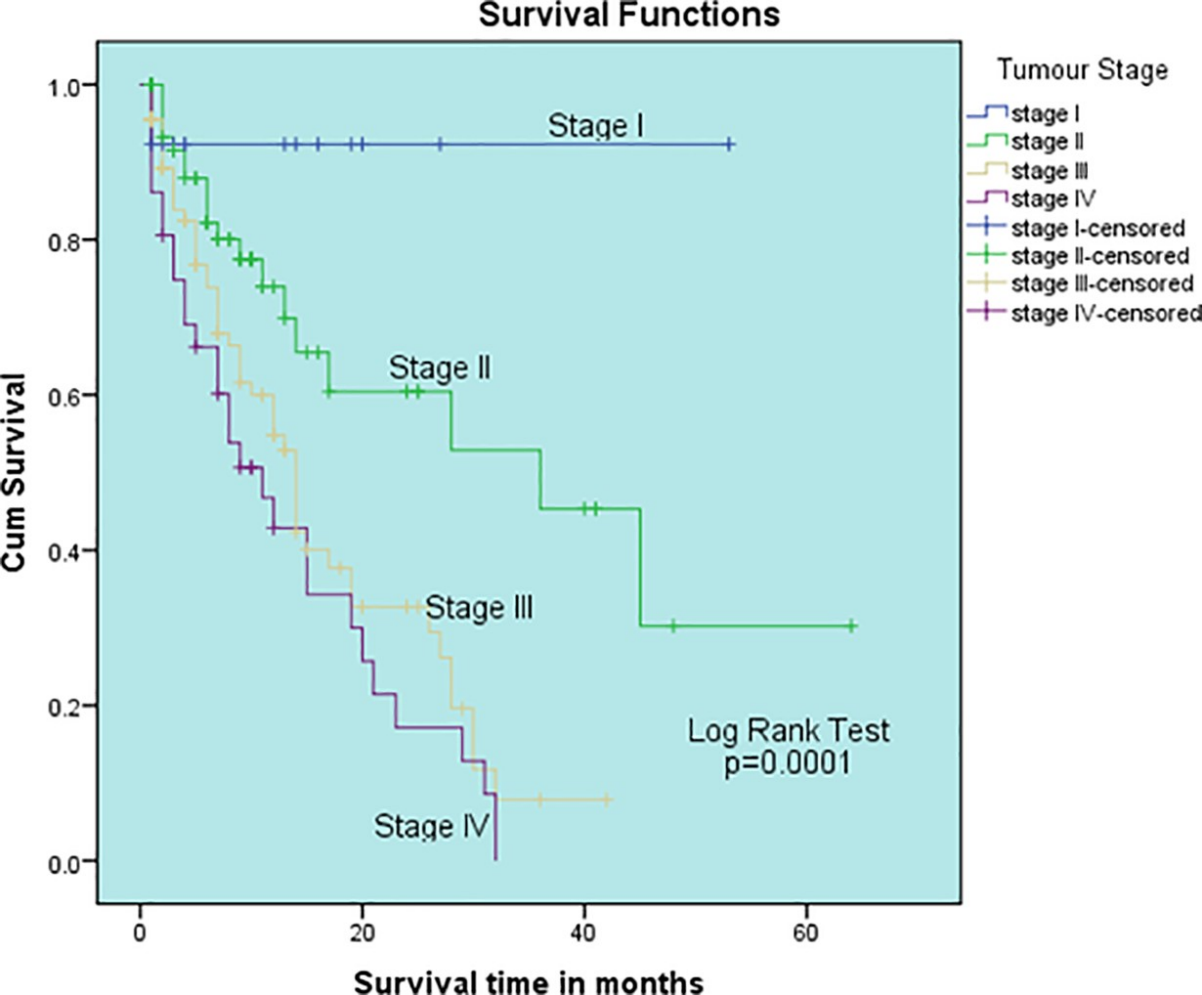
Cumulative survival of CRC based on the cancer stage.

The survival rate was high among patient in stage I: 90% [95% CI (75.5–104.5)], compared to stage II: 34% [95% CI (5.6–62.4)], stage III: 12% [95% CI (11.9–13.1)] and stage IV (0.0%). The differences in survival rates among the different cancer stages were statistically significant (p = 0.0001) **[Fig 3].**

**Table 1** shows the association between socio-demographic and lifestyle characteristics and CRC survival. There was no statistically significant association between survival and age (p = 0.241), gender (p = 0.996), marital status (p = 0.464), presence of comorbidities (p = 0.250), hypertension (p = 0.232), diabetes (p = 0.588), alcohol intake (p = 0.717) and smoking (p = 0.696). Meanwhile, family history was significantly associated with survival (p = 0.036).

As shown in **Table 2**, chemotherapy as a treatment modality was significantly associated with survival (p = 0.0001). The median survival time of patient who underwent chemotherapy was higher (30 months) compared to those who did not undergo chemotherapy (11months). Body mass index (BMI) was also significantly associated with survival (p = 0.036) with underweight patients having the least median survival time (11 months). For the pathological parameters, there was significant difference in the stage of tumour, lymph node metastasis and distance metastasis with survival (p< 0.05). Stage III and IV tumours had low median survival time (Stage III = 14 months, IV = 11months) compared to early stage tumours (36 months). Other parameters such as histological grade (p = 0.332), depth of tumour invasion (p = 0.070) and tumour location (p = 0.405) were not significantly associated with survival.

**Table 3** shows the association of socio-demographic and lifestyle characteristics with survival. Using cox regression analysis, family history was the only variable that was significantly associated with survival on both univariate [HR = 0.37, 95% (0.14–0.99); p = 0.049) and multivariate analysis [HR = 3.44, 95%CI (0.09–0.88)]. Age, gender, marital status, alcoholic intake and smoking history were statistically not associated with survival (p>0.05). The odds of survival decreased as age advanced as shown in the hazard ratios but was not statistically significant (p>0.05). Male gender, being widowed, presence of comorbidities and having hypertension had increased hazard ratios but not statistically significant on both univariate and multivariate analysis.

**Table 4** shows the association between clinical factors and survival using cox regression analysis. In both univariate and multivariate analysis chemotherapy (p = 0.0001) and being underweight (p< 0.05) were significant prognostic factors in colorectal cancer survival. Chemotherapy was associated with high odds of survival (HR: 0.38 (0.25–0.57) whereas having chemo radiotherapy (HR: 3.63(1.05–12.59) and being underweight (HR: 1.74(1.11–2.72) were associated with decrease odds of surviving. Meanwhile, treatment with both chemo and radiotherapy (p = 0.042) were significant prognostic factors for CRC survival after multivariate analysis. Duration of symptoms, surgery, nature of operation and having radiotherapy were not statistically associated with survival (p> 0.05).

The association between pathological factors and survival using cox regression analysis is shown in **Table 5**. Stage of tumour, depth of tumour invasion, lymph node metastasis and distant metastasis were significant prognostic factors on univariate analysis (p< 0.05). Late stage tumours, Stage III [HR: 9.41(1.29–68.58), p = 0.027] and stage IV [HR: 12.89 (1.74–95.24), p = 0.012)] were associated with poor survival. Similarly, T3 [(HR: 3.42 (1.05–11.11), p = 0.041)] and T4 [(HR: 3.51(1.09–11.33) p = 0.036)], N stages, N1 [(HR: 2.65 (1.58–4.43), p = 0.0001)] and N2 [(HR: 2.42(1.24–4.73), p = 0.009)] and M1 [(HR: 12.16(1.37–3.40), P = 0.001)] were associated with poor survival. Tumour location and histological grade were not statistically significant.

## Discussion

Globally, there has been great improvement in colorectal cancer survival over the past decade partly due to early detection and more effective treatments [20]. Howevever, CRC still remains a major cause of mortality in developing countries. This study therefore investigated the survival rate of colorectal cancer and its prognostic factors among patients at the Komfo Anokye Teaching Hospital, in Ghana.

In this study, the overall five year survival rate was 16%, which is extremely lower than the typically reported survival rate in developed countries. A study by sankaranarayanan et al., (2011) on cancer survival in Africa, Asia, and Central America reported that, colorectal cancer survival in Sub-Saharan African countries was extremely poor compared to Asian and central American countries. In sub-Saharan countries like the Gambia and Uganda, the survival was less than 8% compared to 60% survival rate in Korea and this shows the huge variation in cancer survival between these two continents [21]. Lack of modernised infrastructure for cancer care and unavailability of curative treatment for patients were some of the factors identified for the poor cancer survival in Sub Saharan Africa. In Asian countries like China, colorectal cancer patients have 60.8% survival rate after surgery. Studies from other developing countries like Iran reported that the 5-year survival rates of colorectal cancer falls betweeen 27.2% and 61% [22] which are comparatively higher than our current finding.

Mostly, the stage of a cancer at diagnosis influences survival. For colorectal cancer stage,the five-year survival rates varies from 90% for localized cancers, 70% for regional cancers, and 10% for distant metastatic cancers [1]. In this study, the overall survival rates based on CRC TNM staging were 90% for stage I, 34% for stage II, 12% for stage III and 0.0% for stage IV (Fig 3). The difference in survival rate among the different cancer stages using log rank test was statistically significant (p = 0.0001). A study by Al-Ahwal et al., (2013) in Suadi Arabia recorded 63.3% for patients with stage 1 cancers, 50.2% for those with stage 2&3 cancers, and 14.7% for patients stage 4 cancers which are slightly comparable to our findings [23], The lower survival rates observed in this study could be due to the lack of interventions such as screening programmes and public education on cancer prevention, inaccessibility to specialised centers and lack of effective modernised diagnostic techniques for efficient diagnosis and prognosis. Improved life expectancy accompanied with the adoption of sedentary lifestyle and unhealthy dietary habits among Ghanaians have resulted in the rise in the incidence of various cancer including colorectal cancer leading to the high demand for quality cancer care. Studies have also shown that patients mostly present with late stage cancers that are mostly incurable [16] therefore resulting in poorer treatment outcome for patients with colorectal cancers. Late presentation could be due to lack of education on the signs and symptoms of colorectal cancer among the populace, lack of screening programmes for early detection and the fact that most people might be oblivious of the importance of early reporting to hospital for diagnosis and treatment. With colorectal cancer, prognosis is mostly determined by the characteristics of the tumour and some patients related factors. Knowledge of these prognostic factors could help physicians immensely to improve clinical outcomes [24]. Family history was significantly associated with improved survival (p = 0.036) in both the log rank test and the cox regression model (Tables 1 and 3). This is consistent with findings from [25] who reported that patients who have family history of colorectal cancer have overall improved survival compared to those who developed that cancer due to lifestlye factors but not neccesarly due to heredity. The reason could be that, patients with family histroy of the disease are aware of their risk factor, and thus seek early medical intervension and treatments which improves their live expectancy as compared to sporadic cases.

Numerous studies report on the role of patient’s gender as a prognostive factor in colorectal cancer, but in most of these studies, gender palyed no significant role in predicting survival [26–28] which is consistent with findings from our current study. In this study, age was not identified as a prognostic factor for survival. This agrees with several other studies[29–31]. However, some other studies found age as prognostic factor for poor survival in older patients than younger ones. [26, 28, 32], In keeping with Akhood et al., (2011), our study could not approve a significant relationship between survival rate and marital status [33].

Chemotherapy as a treatment modality was significantly related to improved survival whereas having chemo-radiotherapy or radio-chemotherapy was associated with poor survival (Table 4). Most patients with stage III disease are administered chemotherapy after surgery [34]. Such treatment mostly classified as “adjuvant" helps to improve disease outcome by destroying microscopic cancer cells which could have accumulated and developed into larger tumours. This combined therapy has been proven to be effective in enhancing survival by 15–20%. [35]. This explanation supports our finding that chemotherapy is associated with improved survival. A study by Kumar et al., (2015) in Oman found BMI and chemotherapy as independent risk factors of CRC, this supports the findings in this study [36]. There have been conflicting findings on the association between BMI and colorectal cancer survival. A recent meta-analysis reported that being obese before diagnosis of CRC (BMI ≥30 kg/m^2^) was significantly associated with poorer survival [37]. A retrospective study by Tang et al (2016) also found that, being underweight before treatment was associated with an increase risk of death whereas overweight and obesity were favourable prognostic factors for overall survival in metastatic cancer patients [38]. Similarly, our study found that being underweight after diagnosis was significantly associated with poor survival whereas being overweight or obese was more favourable. On the contrary, Boyle et al., (2013) reported, post diagnostic overweight or obesity was associated with poorer survival in colorectal cancer patients [39]. There is a link between obesity and numerous cancer incidences, but in terms of survival, studies have proposed that increasing levels of insulin and insulin-like growth factors as well as increasing insulin resistance in obesity may negatively influence colorectal cancer survival. [40]. It is therefore advisable that colorectal cancer patients maintain a healthy normal weight which will help to improve their survival.

In this present study, the stage of tumor was associated with worse survival (Table 5). This is consistent with several studies [13, 41] that have demonstrated that advanced tumour stage is a prognostic factor associated with poor survival in patients with CRC. Findings from this study showed that, the state of regional lymph node metastasis was a significant prognostic factor for poor suvival, which concurs with findings observed by other studies [42, 43]. Cox proportional hazard model in this current study revealed that, distant metastasis was significantly associated with poor survival (Table 5). This finding is supported by many other studies [44, 45] which also idenfied distance metastasis as a significant factor for poor survival. Other studies have observed a significant relationship between extent of tumour infiltration and prognosis [44, 46], this trend was also observed in this current study. The extent of tumour infiltration into the intestinal wall, lymph nodes and distant organs strongly influences the survival prospects of colorectal cancer patients and also forms the basis for staging as well as treatment options for patients. [9].

Information on some of the study subjects were unavailable because of the retrospective nature of the study. Patients who were diagnosed and treated only at KATH were included in this study, hence this may not be a true reflection of the situation in the entire population, although almost all oncological cases from the Northen and Central sectors of Ghana are refererd to KATH for management. Inspite of these limitations, the study has provided useful information that can help to direct Ghana cancer control strategy inorder to improve cancer survival and help health practitioners in the management of patients with colorectal cancer.

## Conclusion

The survival rate of colorectal cancer is very low in Ghana. Significant clinical and pathological prognostic factors were; family history, chemotherapy, both chemo and radiotherapy, BMI, TNM tumour stage, Depth of tumour invasion, lymph node metastasis, distance metastasis. Therefore, this study highlights the need for intensified public health education to promote awareness about the signs and symptoms of colorectal cancer and comprehensive screening programmes which will greatly improve survival through early detection. Furthermore, molecular studies should be done to identify potential molecular markers for an improved and effective treatment in the Ghanaian population.

## Supporting information

S1 Dataset Excel sheet of dataset on which conclusions of this manuscript were made. (XLSX)

S1 Table Analyses comparing characteristics of patients who were follow-up to those who were not followed. (DOCX)

S1 File STROBE checklist cohort. (DOC)
