# Supplementary material for: Colorectal cancer survival rates in Ghana: A retrospective hospital-based study
Source: PLoS One. 2018 Dec 19;13(12):e0209307. doi: 10.1371/journal.pone.0209307 (PMC6300283; doi:10.1371/journal.pone.0209307)
Supplement: S1 Table — (DOCX) [file pone.0209307.s002.docx]

**Table 1: Comparison of socio-demographic and lifestyle characteristics between patients who were followed and those who were not followed**

| Variables | Patient who were followed | Patients who were not followed | X^2^, df | P-value |
| --- | --- | --- | --- | --- |
|  | n (%) | n (%) |  |  |
| **Age (years)*** |  |  | 9.1, 4 | 0.059 |
| < 40 | 4(12.1) | 29(34.1) |  |  |
| 40-49 | 3(9.1) | 11(12.9) |  |  |
| 50-59 | 8(24.2) | 21(24.7) |  |  |
| 60-69 | 8(24.2) | 10(11.8) |  |  |
| ≥70 | 10(30.3) | 85(72.0) |  |  |
| **Gender**** |  |  |  |  |
| Female | 22(66.7) | 48(56.5%) |  | 0.312 |
| Male | 11(33.3) | 37(43.5) |  |  |
| **Marital Status *** |  |  | 4.9, 3 | 0.178 |
| Single | 2(6.1) | 16(18.8) |  |  |
| Married | 19(57.6) | 40(47.1) |  |  |
| Divorced | 2(6.1) | 11(12.9) |  |  |
| Widowed | 10(30.3) | 18(21.1) |  |  |
| **Family History**** |  |  |  | 0.799 |
| No | 5(15.2) | 8(9.4) |  |  |
| Yes | 28(84.8) | 77(90.6) |  |  |
| **Presence of Comorbidities**** |  |  |  | 0.535 |
| No | 22(66.7) | 83(97.6) |  |  |
| Yes | 11(33.3) | 30(35.3) |  |  |
| **Hypertension **** |  |  |  | 0.338 |
| No | 23(69.7) | 83(97.6) |  |  |
| Yes | 2(6.1) | 2(2.4) |  |  |
| **Diabetes **** |  |  |  | 0.311 |
| No | 32(93.9) | 67(78.8) |  |  |
| Yes | 2(6.1) | 18(21.2) |  |  |
| **Alcoholic intake **** |  |  |  | 0.490 |
| No | 26(78.8) | 60(70.5) |  |  |
| Yes | 7(21.2) | 25(29.4) |  |  |
| **Smoking history **** |  |  |  | 0.051 |
| No | 29(87.8) | 83(97.6) |  |  |
| Yes | 4(12.1) | 2(2.4) |  |  |

***X^2^=Chi-square value, df, * Chi-square test, **Fisher exact test, p<0.05=statistically significant***

**Table 2: Comparison of Clinical parameters between patients who were followed and those who were not followed**

| Variables | Patient who were followed | Patients who were not followed | X^2^, df | P-value |
| --- | --- | --- | --- | --- |
|  | n (%) | n (%) |  |  |
| **Duration of Symptoms (months)*** |  |  | 4.2, 2 | 0.123 |
| < 6 | 11(33.3) | 39(45.9) |  |  |
| 6 to 12 | 11(33.3) | 32(37.6) |  |  |
| > 12 | 11(33.3) | 24(16.5) |  |  |
| **Surgery**** |  |  |  | 0.054 |
| No | 7(21.2) | 35(41.2) |  |  |
| Yes | 26(78.8) | 50(58.8) |  |  |
| **Nature of Operation**** |  |  |  | 0.300 |
| Emergency | 23(69.7) | 26(30.6) |  |  |
| Elective | 10(30.3) | 69(69.4) |  |  |
| **Chemotherapy**** |  |  |  | 0.356 |
| No | 27(81.8) | 58(68.2) |  |  |
| Yes | 6(18.2) | 27(31.8) |  |  |
| **Radiotherapy**** |  |  |  | 0.174 |
| No | 27(81.8) | 58(68.2) |  |  |
| Yes | 6(18.2) | 27(31.8) |  |  |
| **Chemo-radiotherapy**** |  |  |  | 0.803 |
| No | 27(81.8) | 66(77.6) |  |  |
| Yes | 6(18.2) | 19(22.4) |  |  |
| **BMI Categories*** |  |  | 8.2, 3 | 0.846 |
| Underweight | 10(30.3) | 27(31.8) |  |  |
| Normal | 15(45.5) | 37(43.5) |  |  |
| Overweight | 6(18.2) | 12(14.1) |  |  |
| Obese | 2(6.1) | 9(10.6) |  |  |

***X^2^=Chi-square value, df, * Chi-square test, **Fisher exact test, p<0.05=statistically significant***

**Table 3: Comparison of pathological parameters between patients who were followed and those who were not followed**

| **Variables** | Patients who were followed | Patients who were not followed | X^2^, df | P-value |
| --- | --- | --- | --- | --- |
|  | n (%) | n (%) |  |  |
| **Tumour Location*** |  |  | 8.9, 4 | 0.064 |
| Colon | 11(33.3) | 27(31.8) |  |  |
| Rectum | 19(57.7) | 38(44.7) |  |  |
| Anorectum | 0(0.0) | 14(16.5) |  |  |
| Anal | 1(3.0) | 5(5.9) |  |  |
| More than one site | 2(6.0) | 1(1.2) |  |  |
| **Histological Grade*** |  |  | 0.7, 3 | 0.875 |
| Well differentiated | 11(33.3) | 22(27.8) |  |  |
| Moderately differentiated | 15(45.5) | 37(46.8) |  |  |
| Poorly differentiated | 4(12.1) | 9(11.4) |  |  |
| Undifferentiated | 3(9.1) | 11(13.9) |  |  |
| **Tumour Stage*** |  |  | 5.6, 3 | 0.132 |
| Stage 1 | 4(12.1) | 20(23.6) |  |  |
| Stage II | 15(45.5) | 19(22.4) |  |  |
| Stage III | 7(21.2) | 20(23.6) |  |  |
| Stage IV | 7(21.2) | 19(22.4) |  |  |
| **Depth of Tumour Invasion*** |  |  | 3.4, 2 | 0.185 |
| T2 | 4(12.1) | 22(25.9) |  |  |
| T3 | 17(51.5) | 31(36.5) |  |  |
| T4 | 12(36.4) | 32(37.6) |  |  |
| **Lymph Node Metastasis*** |  |  | 5.8, 2 | 0.056 |
| N0 | 30(90.9) | 61(71.8) |  |  |
| N1 | 3(9.1) | 15(17.7) |  |  |
| N2 | 0(0.0) | 9(10.5) |  |  |
| **Distant Metastasis**** |  |  |  | 0.442 |
| M0 | 32(96.9) | 77(90.6) |  |  |
| M1 | 1(3.1) | 8(9.4) |  |  |

***X^2^=Chi-square value, df, * Chi-square test, **Fisher exact test, p<0.05=statistically significant***
