# Supplementary material for: Colorectal cancer survival rates in Ghana: A retrospective hospital-based study
Source: PLoS One. 2018 Dec 19;13(12):e0209307. doi: 10.1371/journal.pone.0209307 (PMC6300283; doi:10.1371/journal.pone.0209307)
Supplement: S1 File — (DOC) [file pone.0209307.s003.doc]

STROBE Statement—Checklist of items that should be included in reports of ***cohort studies***

|  | Item No | Recommendation |
| --- | --- | --- |
| **Title and abstract** | 1 | (*a*) Indicate the study’s design with a commonly used term in the title or the abstract  **Colorectal Cancer Survival Rates in Ghana: A Retrospective Cohort Study** |
|  |  | (*b*) Provide in the abstract an informative and balanced summary of what was done and what was found  **Background:** Despite medical interventions, the overall clinical outcomes and survival of colorectal cancer remains very poor for most patients in developing countries.  **Objective**: To investigate the survival rate of colorectal cancer and its prognostic factors among patients at Komfo Anokye Teaching Hospital, Ghana.  **Design:** Retrospective Cohort Study  **Setting:** Komfo Anokye Teaching Hospital, in Ghana.  Patients: All colorectal cancer patients diagnosed and receiving care at KATH in Ghana from 2009 to 2016. Patients with medical records showing complete clinical examination, indicating the presence of malignant tumour in the large bowel were included. Whereas those with other large bowel conditions and histopathological confirmed non-malignant tumours were excluded.  **Measurements**: The survival graphs were obtained using the Kaplan–Meier method and compared by the Log-rank test. Cox regression analysis was used to assess prognostic factors. All analyses were performed by SPSS version 22.  **Results:** The median survival time was 15 months 95% CI (11.79-18.21). The survival rates at the 1^st^, 2^nd^, 3^rd^, 4^th^ and 5^th^ years were 64% 95% CI (56.2-71.1), 40% 95% CI (32.2-50.1), 21% 95% CI (11.4-30.6) 16% 95% CI (8.9-26.9) and 16% 95% CI (7.3-24.9) There was a significant difference in the survival rate of colorectal cancer according to the different stages (p=0.0001). Family history [HR=3.44, p=0.029)], Chemotherapy [HR= 0.23, p=<0.0001)], BMI [HR=1.78, p=0.017)] and both chemo/radiotherapy (HR= 3.63, p=0.042)] were the significant social and clinical factors influencing the overall survival. Pathological factors such as TNM tumour stage (p=0.012), depth of tumour invasion (p=0.036), lymph node metastasis (p=0.0001), and distance metastasis (p=0.001) were significantly associated with overall survival.  **Conclusions:**  The study has clearly demonstrated that survival rate for CRC patients at KATH, Ghana is very low in a 5 years period. This is influenced by significant number of clinical and pathological prognostic factors. Identification of prognostic factors would be a primary basis for early prediction and treatment of patients with colorectal cancer. |
| Introduction | | |
| Background/rationale | 2 | Explain the scientific background and rationale for the investigation being reported  Globally, colorectal cancer is one of the commonest cancers and in the western countries; it is the second leading cause of cancer mortality. . Survival of colorectal cancer has improved dramatically over the last decade as a result of the invention of new drugs and targeted therapies. . However, enormous disparities in colorectal cancer survival exist within regions and across the global . These differences are not easily understood, although most of the disparities in CRC survival can be attributed to variation in the accessibility to treatment and diagnostics . Also the difference in survival rates observed in various clinical trials maybe due to the variations in patient’s characteristics and prognostic factors . In addition, molecular analyses performed so far indicates that the pathogenesis of all CRCs varies at different stage tumours. Even for individual patients with same stage tumours, response to treatment and long term prognosis varies.  Over the past years, several research groups have suggested numerous factors associated with the survival of CRC patients.. However, the extent of tumour infiltration to the bowel wall, adjacent lymph node metastases and distant metastasis are the major contributing factors . Although, various studies have reported a strong correlation between colorectal cancer stage and its prognosis, it has also been argued in other studies that the prognosis for a patient with colorectal cancer is much influenced by factors relating to patients characteristics and the tumour but not just the anatomical extension of the tumour.  Additionally, other studies have also showed that the initial treatment administered, body mass index (BMI), marital status, tumour grade, tumour size and pathologic stage of tumour are significantly associated with the survival of CRC patients . Recent studies have shown that the survival of CRC in sub-Saharan African is very low due to late presentations and lack of modern specialized systems for treatment . In Ghana, the number of new cases of colorectal cancer has increased by 8- fold per year from an average of 4.1 new cases in 1960s to an average of 32.6 new cases currently . In 2010, Dakubo *et al* reported a crude incidence rate of 11.18 per 100,000 populations in both sexes. Moreover, Laryea et al., (2014) reported a crude incidence and age standardized incidence of 0.1 and 0.3 per 100,000 population [[17]](#_ENREF_17). Some studies have identified other factors such as helicobacter pylori infection, the dietary component of red meat, beef, lamb, pork and veal and its processed varieties as the predominant risk factors in Ghana . There is paucity of data on the survival rate of CRC as well as its associated factors in Ghana. Knowledge of prognostic factors in our population will be the foundation for planning treatment and predicting the outcome of patients with colorectal cancer. It is thus, against this background that this study investigated the survival rate of colorectal cancer and its prognostic factors among patients at Komfo Anokye Teaching Hospital, Ghana. |
| Objectives | 3 | State specific objectives, including any prespecified hypotheses  **SPECIFIC OBJECTIVES**:  1. To estimate the five-year survival rate of colorectal cancer from 2009 to 2015.  2.To evaluate the relationship between social, clinical and pathological parameters and overall survival (OS) in colorectal cancer patients.  3. To investigate the survival rate of colorectal cancer and its prognostic factors among patients at Komfo Anokye Teaching Hospital, Ghana.  **HYPOTHESIS:**  There is no association between the clinico-pathological factors and overall survival of colorectal cancer. |
| Methods | | |
| Study design | 4 | Present key elements of study design early in the paper  This was a retrospective cohort study where all colorectal cancer cases presented to the Surgical and Oncological Department of Komfo Anokye Teaching Hospital (KATH) were reviewed. The medical records of all CRC patients managed at the Hospital from 2009 to 2015 were retrieved from the Medical records unit of the Surgery and Oncology Department. The records were analysed for information on demographics, clinical and pathological variables. Follow up information was based on patients visit after their diagnosis. |
| Setting | 5 | Describe the setting, locations, and relevant dates, including periods of recruitment, exposure, follow-up, and data collection  This study was conducted at the Surgical and Oncological (S&O) Department of the Komfo Anokye Teaching Hospital in Ghana. Komfo Anokye Teaching Hospital (KATH) is the second largest and a referral teaching hospital located in Kumasi, the regional capital of the Ashanti region in Ghana. The region has an average total population of 4,780,380 (Ghana Statistical Service, 2010). Data collection was started in November 2015 and ended in April 2016. Patients were contacted during their follow-up visits to the hospital within this period and those who could not report for review were contacted via telephone. |
| Participants | 6 | (*a*) Give the eligibility criteria, and the sources and methods of selection of participants. Describe methods of follow-up  Patient’s medical records showing complete clinical examination, indicating the presence of malignant tumour in the large bowel were included. Cases that were excluded included patients with large bowel conditions other than colorectal cancer and histopathologically confirmed non-malignant tumours.  Patients were contacted during their follow-up visits to the hospital and those who could not report for review in the hospital were contacted via telephone. Deaths of subjects were confirmed via contact with their families and relatives. Survival periods were calculated from the date of diagnosis to the date of either last follow-up or death. Patients alive at the end of the follow-up and those lost to follow-up were censored either at the last contact or at death. |
|  |  | (*b*) For matched studies, give matching criteria and number of exposed and unexposed |
| Variables | 7 | Clearly define all outcomes, exposures, predictors, potential confounders, and effect modifiers. Give diagnostic criteria, if applicable.  Only patients with colorectal cancer cases were included. All other large bowl conditions were excluded. In the statistical analysis, factors such as age and presence of comorbidities were taking into consideration as potential confounding factors.  Outcome; Dead or alive (Survival)  Exposure; Colorectal cancer  Predictors; Social factors, Clinical factors and pathological factors  Potential confounders: Age, presence of comorbidities, smoking, alcohol intake  Effect modifiers; Surgery, type of therapy either chemotherapy or both chemo and radiotherapy |
| Data sources/ measurement | 8* | For each variable of interest, give sources of data and details of methods of assessment (measurement). Describe comparability of assessment methods if there is more than one group.  Survival periods were calculated from the date of diagnosis to the date of either last follow-up or death. Patients alive at the end of the follow-up and those lost to follow-up were censored either at the last contact or at death. BMI was calculated based on the last recorded weight and height in the patients’ medical records.  Patients survival or death status was confirmed via telephone call or visit of patient to the hospital. All other information in relation to social factors, clinical and pathological were obtained from the patients’ medical records. |
| Bias | 9 | Describe any efforts to address potential sources of bias  Types of possible bias; Information bias.  Some data were censored in the survival analysis in other to prevent bias in the calculation of survival time. |
| Study size | 10 | Explain how the study size was arrived at  The number of cases of colorectal cancer within the study period determined the study size. |
| Quantitative variables | 11 | Explain how quantitative variables were handled in the analyses. If applicable, describe which groupings were chosen and why  Quantitative variables were normalised and analysed using SPSS. |
| Statistical methods | 12 | Describe all statistical methods, including those used to control for confounding  Survival analysis was done using Kaplan–Meier method and the differences in patient survival periods were determined by employing the log-rank test. To determine the prognostic factors for survival, all variables were tested for their relationship in the Cox-regression model. Proportional hazard (PH) assumptions were initially tested for each model [Table 3, 4 and 5] based on the scaled Schoenfeld residuals. The PH test was not significant for each of the covariates in each model and the global Schoenfeld test (GST) was not statistically significant for each model [Table 3; GST: p=0.481, Table 4; GST: p=0.186, Table 5; GST: p=0.216], we therefore assumed the proportional hazards. Multicollinearity test was done for covariates in each model, and the variation inflation factor (VIF) value obtained for covariates in each model was 1-5, suggesting that there is no multicollinearity. Multivariable Cox regression analysis was carried using the force entry procedure. The Chi-square value obtained for the regression model for Table 4 (χ^2^ =42.7, p<0.0001) and Table 5 (χ^2^ = 28.8, p=0.017) were statistically significant, however Chi-square value for regression model for Table 3 did not show significance (χ^2^ =15.8, p=0.328). A p < 0.05 was accepted as statistically significant. |
|  |  | (*b*) Describe any methods used to examine subgroups and interactions |
|  |  | (*c*) Explain how missing data were addressed  Patients with incomplete data were excluded from the study |
|  |  | (*d*) If applicable, explain how loss to follow-up was addressed  Patients were contacted during their follow-up visits to the hospital and those who could not report for review in the hospital were contacted via telephone. Deaths of subjects were confirmed via contact with their families and relatives. Survival periods were calculated from the date of diagnosis to the date of either last follow-up or death. Patients alive at the end of the follow-up and those lost to follow-up were censored either at the last contact or at death. |
|  |  | (*e*) Describe any sensitivity analyses |
| Results | | |
| Participants | 13* | (a) Report numbers of individuals at each stage of study—eg numbers potentially eligible, examined for eligibility, confirmed eligible, included in the study, completing follow-up, and analysed.  In all 221 patients were recruited for this study. Among the patients, 33 were alive, 103 died and 85 were lost to follow up. |
|  |  | (b) Give reasons for non-participation at each stage |
|  |  | (c) Consider use of a flow diagram  Flow diagram have submitted as Figure 1 |
| Descriptive data | 14* | (a) Give characteristics of study participants (eg demographic, clinical, social) and information on exposures and potential confounders  Demographic, clinical and social information of study participants include age, gender, marital status, family history, presence of comorbidities, diabetes, and alcohol intake history of smoking.  clinical and pathological variables including histological type, grade of tumour and TNM staging |
|  |  | (b) Indicate number of participants with missing data for each variable of interest  n/a |
|  |  | (c) Summarise follow-up time (eg, average and total amount)  The study period includes patients diagnosed from 2009 to 2015 ie  The median survival time was 15 months 95% CI (11.79-18.21). |
| Outcome data | 15* | Report numbers of outcome events or summary measures over time |
| Main results | 16 | (*a*) Give unadjusted estimates and, if applicable, confounder-adjusted estimates and their precision (eg, 95% confidence interval). Make clear which confounders were adjusted for and why they were included |
|  |  | (*b*) Report category boundaries when continuous variables were categorized |
|  |  | (*c*) If relevant, consider translating estimates of relative risk into absolute risk for a meaningful time period |
| Other analyses | 17 | Report other analyses done—eg analyses of subgroups and interactions, and sensitivity analyses |
| Discussion | | |
| Key results | 18 | Summarise key results with reference to study objectives  In this study, we found that, the overall five-year survival rate of colorectal cancer at KATH in Ghana is 16% which is very low and conform to what has been stipulated in most studies that the survival rate of colorectal cancer in developing countries is low. We also identified that this low survival rate is influenced by significant number of social, clinical and pathological prognostic factors. These factors include cancer stage, family history, chemotherapy, BMI and both chemo and radiotherapy. Pathological factors such as TNM tumour stage, depth of tumour invasion, lymph node metastasis and distance metastasis were also significantly associated with survival. These factors may serve as a primary basis for early prediction and treatment of patients with colorectal cancer. |
| Limitations | 19 | Discuss limitations of the study, considering sources of potential bias or imprecision. Discuss both direction and magnitude of any potential bias  Information on some of the study subjects were unavailable because of the retrospective nature of the study. Patients who were diagnosed and treated only at KATH were included in this study, hence this may not be a true reflection of the situation in the entire population, although almost all oncological cases from the Northern and Central sectors of Ghana are referred to KATH for management. Despite these limitations, the study has provided useful information that can help to direct Ghana cancer control strategy in order to improve cancer survival and help health practitioners in the management of patients with colorectal cancer. |
| Interpretation | 20 | Give a cautious overall interpretation of results considering objectives, limitations, multiplicity of analyses, results from similar studies, and other relevant evidence  Globally, there has been great improvement in colorectal cancer survival over the past decade partly due to early detection and more effective treatments . However, CRC remains a major cause of mortality in developing countries. This study therefore investigated the survival rate of colorectal cancer and its prognostic factors among patients at the Komfo Anokye Teaching Hospital, in Ghana.  In this study, the overall five-year survival rate was 16%, which is extremely lower than the typically reported survival rate in developed countries. A study by sankaranarayanan et al., (2011) on cancer survival in Africa, Asia, and Central America reported that, colorectal cancer survival in Sub-Saharan African countries was extremely poor compared to Asian and central American countries. In sub-Saharan countries like the Gambia and Uganda, the survival was less than 8% compared to 60% survival rate in Korea and this shows the huge variation in cancer survival between these two continents [[21]](#_ENREF_21). Lack of modernised infrastructure for cancer care and unavailability of curative treatment for patients were some of the factors identified for the poor cancer survival in Sub Saharan Africa. In Asian countries like China, colorectal cancer patients have 60.8% survival rate after surgery. Studies from other developing countries like Iran reported that the 5-year survival rates of colorectal cancer falls between 27.2% and 61% which are comparatively higher than our current finding.  Mostly, the stage of a cancer at diagnosis influences survival. For colorectal cancer stage, the five-year survival rates varies from 90% for localized cancers, 70% for regional cancers, and 10% for distant metastatic cancers . In this study, the overall survival rates based on CRC TNM staging were 90% for stage I, 34% for stage II, 12% for stage III and 0.0% for stage IV (Figure 2). The difference in survival rate among the different cancer stages using log rank test was statistically significant (p=0.0001). A study by Al-Ahwal et al., (2013) in Suadi Arabia recorded 63.3% for patients with stage 1 cancers,, 50.2% for those with stage 2&3 cancers, and 14.7% for patients stage 4 cancers which are slightly comparable to our findings  [[23]](#_ENREF_23), The lower survival rates observed in this study could be due to the lack of interventions such as screening programs and public education on cancer prevention, inaccessibility to specialized centers and lack of effective modernized diagnostic techniques for efficient diagnosis and prognosis. Improved life expectancy accompanied with the adoption of sedentary lifestyle and unhealthy dietary habits among Ghanaians have resulted in the rise in the incidence of various cancer including colorectal cancer leading to the high demand for quality cancer care. Studies have also shown that patients mostly present with late stage cancers that are mostly incurable therefore resulting in poorer treatment outcome for patients with colorectal cancers. Late presentation could be due to lack of education on the signs and symptoms of colorectal cancer among the populace, lack of screening programmes for early detection and the fact that most people might be oblivious of the importance of early reporting to hospital for diagnosis and treatment. With colorectal cancer, prognosis is mostly determined by the characteristics of the tumour and some patients related factors. Knowledge of these prognostic factors could help physicians immensely to improve clinical outcomes . Family history was significantly associated with improved survival (p=0.036) in both the log rank test and the cox regression model (Table 1 and 3). This is consistent with findings from who reported that patients who have family history of colorectal cancer have overall improved survival compared to those who developed that cancer due to lifestyle factors but not necessarily due to heredity .The reason could be that, patients with family history of the disease are aware of their risk factor, and thus seek early medical intervention and treatments which improves their live expectancy as compared to sporadic cases.  Numerous studies report on the role of patient’s gender as a prognostic factor in colorectal cancer, but in most of these studies, gender played no significant role in predicting survival which is consistent with findings from our current study.  In this study, age was not identified as a prognostic factor for survival. This agrees with several other studies. However, some other studies found age as prognostic factor for poor survival in older patients than younger ones. , In keeping with Akhood et al., (2011), our study could not approve a significant relationship between survival rate and marital status [[33]](#_ENREF_33).  Chemotherapy as a treatment modality was significantly related to improved survival whereas having chemo-radiotherapy or radio-chemotherapy was associated with poor survival (Table 4). Most patients with stage III disease are administered chemotherapy after surgery . Such treatment mostly classified as “adjuvant" helps to improve disease outcome by destroying microscopic cancer cells which could have accumulated and developed into larger tumours. This combined therapy has been proven to be effective in enhancing survival by 15-20%. . This explanation supports our finding that chemotherapy is associated with improved survival. A study by Kumar et al., (2015) in Oman found BMI and chemotherapy as independent risk factors of CRC, this supports the findings in this study [[36]](#_ENREF_36). There have been conflicting findings on the association between BMI and colorectal cancer survival. A recent meta-analysis reported that being obese before diagnosis of CRC (BMI ≥30 kg/m^2^) was significantly associated with poorer survival . A retrospective study by Tang et al (2016) also found that, being underweight before treatment was associated with an increased risk of death whereas overweight and obesity were favourable prognostic factors for overall survival in metastatic cancer patients [[38]](#_ENREF_38). Similarly, our study found that being underweight after diagnosis was significantly associated with poor survival whereas being overweight or obese was more favourable. On the contrary, Boyle et al., (2013) reported, post diagnostic overweight or obesity was associated with poorer survival in colorectal cancer patients  [[39]](#_ENREF_39). There is a link between obesity and numerous cancer incidences, but in terms of survival, studies have proposed that increasing levels of insulin and insulin-like growth factors as well as increasing insulin resistance in obesity may negatively influence colorectal cancer survival. . It is therefore advisable that colorectal cancer patients maintain a healthy normal weight which will help to improve their survival.  In this present study, the stage of tumor was associated with worse survival (Table 5). This is consistent with several studies that have demonstrated that advanced tumor stage is a prognostic factor associated with poor survival in patients with CRC. Findings from this study showed that, the state of regional lymph node metastasis was a significant prognostic factor for poor survival, which concurs with findings observed by other studies . Cox proportional hazard model in this current study revealed that, distant metastasis was significantly associated with poor survival (Table 5). This finding is supported by many other studies which also identified distance metastasis as a significant factor for poor survival. Other studies have observed a significant relationship between extent of tumor infiltration and prognosis , this trend was also observed in this current study. The extent of tumour infiltration into the intestinal wall, lymph nodes and distant organs strongly influences the survival prospects of colorectal cancer patients and also forms the basis for staging as well as treatment options for patients. . |
| Generalisability | 21 | Discuss the generalisability (external validity) of the study results  Findings from this study can not be generalised since only patients who were diagnosed and treated at one hospital (KATH) were included, hence this may not be a true reflection of the situation in the entire population. |
| Other information | | |
| Funding | 22 | Give the source of funding and the role of the funders for the present study and, if applicable, for the original study on which the present article is based  The authors declare that there is no conflict of interests regarding the publication of this paper. |

*Give information separately for exposed and unexposed groups.

**Note:** An Explanation and Elaboration article discusses each checklist item and gives methodological background and published examples of transparent reporting. The STROBE checklist is best used in conjunction with this article (freely available on the Web sites of PLoS Medicine at http://www.plosmedicine.org/, Annals of Internal Medicine at http://www.annals.org/, and Epidemiology at http://www.epidem.com/). Information on the STROBE Initiative is available at http://www.strobe-statement.org.
